# The Biology of Aging and Cancer: A Brief Overview of Shared and Divergent Molecular Hallmarks

**DOI:** 10.14336/AD.2017.0103

**Published:** 2017-10-01

**Authors:** Jan R. Aunan, William C Cho, Kjetil Søreide

**Affiliations:** ^1^Gastrointestinal Translational Research Unit, Molecular Lab, Stavanger University Hospital, Stavanger, Norway; ^2^Department of Gastrointestinal Surgery, Stavanger University Hospital, Stavanger, Norway; ^3^Department of Clinical Oncology, Queen Elizabeth Hospital, Kowloon, Hong Kong; ^4^Department of Clinical Medicine, University of Bergen, Bergen, Norway

**Keywords:** aging, cancer, genomic instability, epigenetic, telomere, stem cells, genomic instability, metabolism

## Abstract

Aging is the inevitable time-dependent decline in physiological organ function and is a major risk factor for cancer development. Due to advances in health care, hygiene control and food availability, life expectancy is increasing and the population in most developed countries is shifting to an increasing proportion of people at a cancer susceptible age. Mechanisms of aging are also found to occur in carcinogenesis, albeit with shared or divergent end-results. It is now clear that aging and cancer development either share or diverge in several disease mechanisms. Such mechanisms include the role of genomic instability, telomere attrition, epigenetic changes, loss of proteostasis, decreased nutrient sensing and altered metabolism, but also cellular senescence and stem cell function. Cancer cells and aged cells are also fundamentally opposite, as cancer cells can be thought of as hyperactive cells with advantageous mutations, rapid cell division and increased energy consumption, while aged cells are hypoactive with accumulated disadvantageous mutations, cell division inability and a decreased ability for energy production and consumption. Nonetheless, aging and cancer are tightly interconnected and many of the same strategies and drugs may be used to target both, while in other cases antagonistic pleiotrophy come into effect and inhibition of one can be the activation of the other. Cancer can be considered an aging disease, though the shared mechanisms underpinning the two processes remain unclear. Better understanding of the shared and divergent pathways of aging and cancer is needed.

Aging is an inevitable time-dependent decline in physiological organ function and is a major risk factor for one of the most significant causes of human morbidity and mortality, namely cancer. According to the US National Cancer Institute’s Surveillance Epidemiology and End Results (SEER) Database, 43% of men and 38% of women will develop an invasive cancer over a lifetime. Among these, 23% of men and 19 % of women will die from cancer. More than half of cancers occur in individuals older than 70 [1].

Improvements in healthcare and healthier nutrition have increased the average life expectancy over the past century. According to the World Health Organization (WHO) life expectancy is now exceeding 80 years in most developed countries. As the population is aging, cancer is becoming an ever more important health burden worldwide.

The underlying mechanism in both cancer and aging is the time dependent accumulation of cellular damage [2]. Cancer and aging may seem like opposite processes - cancer cells have the ‘gain of function and fitness’ whereas aging cells are characterized by a ‘loss of function and fitness’ [2]. However, the two traits do share many common characteristics (Table 1), for which a comparative review will be presented.

**Table 1:** Hallmarks that are either shared or divergent in aging and cancer.

Feature	Aging	Cancer
**Genomic instability**	Increased	Increased
**Telomere attrition**	Shortened telomeres	Shortened telomeres but telomerase activation
**Epigenetic alteration:**		
DNA methylation	Global hypomethylation	Hyper- of tumor suppressors and hypo- of oncogenes
Histone modification Non-coding DNA	Complex miRNA deregulation; for example, miR17-92 downregulation	Complex miRNA deregulation, for example, miR-17-92 upregulation
**Proteostasis:**		
Chaperoning	Impaired	Augmented
Proteasome activity	Impaired	Augmented
Autophagy-lysosome activity	Impaired	Augmented
**Deregulated nutrient sensing**	Inhibition of insulin and mTOR signaling increase lifespan	Inhibition of insulin and mTOR signaling is antineoplastic
**Cellular senescence**	Increased	Prevalent in premalignant tumors but evaded in fully malignant tumors
**Stem cell**	Exhausted	Potential nidus for tumorigenesis

## 1. Genomic instability

One of the immediate common hallmarks to both aging and cancer is the occurrence of genomic instability. The human DNA is vulnerable to mutagens such as exogenous radiation and endogenous free radicals, to which we are exposed constantly over a life-time. Indeed, cells in the human body undergo cell-division billions of times during which the DNA is replicated, each time with the risk of introducing or suffering mutational events. Most of these inevitable mutations are harmless and the majority is corrected by the DNA repair system. However, a certain degree of accumulated DNA damage occurs with time [3]. The normal mutation rate after repair in the human genome is about one mutation per billion bases per division, witch means that a 1,000 bp gene has a one in a million chance of a single mutation per cell division[4]. Genomic instability in key regulator functions is the most prominent "enabling hallmark" that leads cancer cells to acquire many of the driving cancer traits, such as self sufficiency in growth signals and increased metastatic potential [5]. Genomic instability is a characteristic of almost all human cancers and the rate of mutation is higher than in normal cells in order to acquire all the mutations needed for tumorigenesis [6]. Indeed, with increasing knowledge and lessons learned from whole genome sequencing of numerous cancers, the mutation rate is known to be overall high yet with different rates according to tumor types and site, with some tumors being labeled hypermutated [7].

### Lessons from hereditary syndromes

Different mutations in the DNA repair machinery lead to hereditary cancers syndromes such as hereditary non-polyposis colorectal cancer (HNPCC)[8], breast and ovarian cancer with hereditary mutations in BRCA1 and BRCA2 [9] and familial adenomatous polyposis with an almost 100% lifetime risk of colorectal cancer[10]. These are just a few examples of a large group of such cancer syndromes resulting from mutations in the DNA repair machinery, giving rise an increased mutational rate and genomic instability. Mutations in the DNA repair machinery can also cause progeroid premature aging syndromes of which there are many [11]. Werner syndrome and Bloom syndrome are progeroid syndromes, both of which are caused by mutations in RecQ helicases involved in repair of double stranded breaks and telomere maintenance [12, 13]. They manifest with cancers at a very young age, premature aging signs (greying of hair, loss of organ function and reserve) and a significantly reduced lifespan [14]. Cockayne syndrome (CS) and Xeroderma Pigmentosum (XP) are two other examples of progeroid syndromes, both a result of mutations in the nucleotide excision repair system (NER). The NER mutation in CS affects only actively transcribed genes (TCR-transcription coupled repair) whereas the damage in XP affects the whole genome (GGR-global genomic repair) [15]. They both manifest with features of premature aging, but interestingly only XP manifest with increased susceptibility to skin cancer [15]. The mechanism behind is still unknown, but it has been speculated that it may involve apoptosis of mutated cells in CS [16].

**Figure 1.**
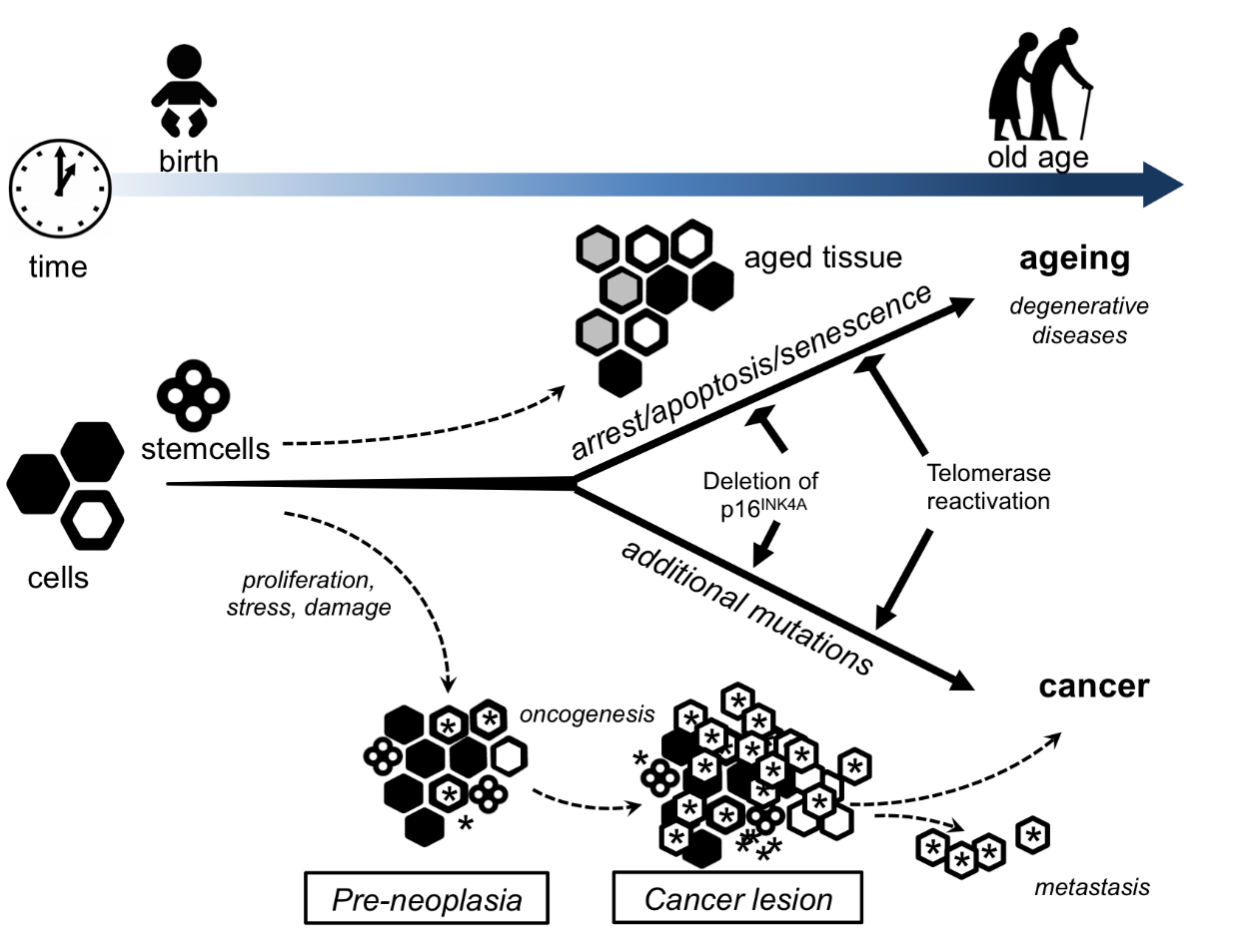
Lifelong interplay between stem cells in aging and cancer A simplified model that views aging and cancer from the perspective of alterations within the stem and progenitor cell pool. Over the lifespan of an organism, long-lived cells (such as stem cells) accumulate DNA damage from a number of stresses including intracellular oxidants generated from normal metabolism. The default pathway for such damaged stem cells is to undergo growth arrest, apoptosis or senescence. As more and more stem cells withdraw from the proliferative pool, there is a decrease in the overall number and/or functionality of both stem and progenitor cells. This decrease predisposes the organism to impaired tissue homeostasis and regenerative capacity and could contribute to aging and age-related pathologies. Presumably, some rare cells can escape from this normal default pathway by acquiring additional mutations that allow them to continue to proliferate even in the setting of damaged DNA. These proliferating but damaged cells might provide the seeds for future malignancies. In this scenario, both cancer and aging result primarily from accumulating damage to the stem and progenitor cell compartment. Mutations that allow stem cells to continue to proliferate in the setting of normal growth arrest signals such as DNA damage (for example, loss of p16^INK4a^ or reactivation of telomerase) would temporarily expand the stem cell pool and hence delay age-related pathologies. Over the long term, these mutations would also increase the likelihood of cancer. During normal aging, stem cells accumulate damage and subsequent stress-dependent changes, for example, de-repression of the *CDKN2a* (p16*^INK4a^*^/^*^ARF^*) locus or telomere shortening. This leads to the increasing abundance of senescent cells (large hexagonal cells) within differentiated tissues. Preneoplastic leasions, arising directly from stem cells or from more committed cells, undergo rapid proliferation (small cells marked with asterisks). These pre-malignant tumor cells rapidly accumulate damage, in part owing to the presence of oncogenes, leading to a higher proportion of tumor cells becoming senescent (cells marked as hexagons filled with white color). Tumor progression to full malignancy is favoured when tumor cells acquire mutations that impair the senescence program (for example, mutations in *Trp53* or *CDKN2a*). Illustration is modified and based upon Finkel T, Serrano M, Blasco MA. The common biology of cancer and aging. Nature. 2007 Aug 16;448(7155):767-74. Copyright © 2007.

### Genomic instability in aging cells

Maintenance of genomic stability appears to be a core function to prevent cancer development as well as aging processes. Genomic instability and mutations may contribute to aging in several ways, ranging from small point mutations to large translocations and deletions. In somatic cells, it can give rise to disruption of the phenotype through alterations in the protein coding sequence but probably more common is the alteration of regulatory sequences leading eventually to progressive decline of organ function due to alterations of the proteome and homeostasis [17, 18]. Indeed there is a large variation in gene expression among cells in the same tissue with age, and in an experiment on mice cardiomyocytes in young and old individuals the variation was significant in all genes tested nuclear genes which included 7 housekeeping genes, 3 heart specific genes and 2 protease genes [18]. The increased gene heterogeneity with age seems to be tissue specific, where for instance small intestinal cells accumulated mainly point mutations and cardiomyocytes also carried large rearrangements [19] This gene heterogeneity is proposed to lead to stochastic deregulation of gene expression among neighboring cells and lead to aging[18]. Genetic instability can also cause aging if the damage is large enough to cause apoptosis or senescence of stem cells leading to the depletion of division competent cells [17].

### Rivaling processes

Cancer and aging are in a way rivaling processes [18]. On the one hand, cancer is the result of advantageous mutations that confers an advantage to the neoplastic cell when it comes to growth and metastasis (Fig. 1). On the other hand, aging is the result of harmful mutations detrimental to the cells physiology or damage leading to senescence, apoptosis and eventually depletion of stem cells [17, 20, 21]. The idea of antagonistic pleiotrophy comes to play where high levels of cell cycle gatekeeper proteins - such as p53 - results in senescence and apoptosis protecting against harmful mutations and tumorigenesis, while low levels allows cells with tumorigenic mutations to propagate and promote cancer [17, 21]. Genomic instability thus reflects the wear and tear on cells over time, leading to controlled cell death and loss of tissue in aging. Yet, it also represents the stochastic risk of accumulating changes that fosters uncontrolled cell division and expansion in cancer, evidenced by the higher lifetime cancer risk of tissues requiring many cell divisions for homeostasis, indicating that the number one cause of tissue cancer variation is just "bad luck" [22]. A deeper understanding of genomic instability may thus help understand its relation to both aging as a process and the role in carcinogenesis with potential for therapeutic interventions in both areas.

## 2. *Telomere attrition*

Most normal human cells have a limited timespan before they undergo controlled cell death (apoptosis). Cells can only undergo a limited number of cell divisions before the cap ends of the DNA is used up and the cell enters an apoptotic program. Apoptosis prevents cells from living eternally and accumulating mutations as the older cells get replaced. The cap end of the DNA is the telomeres, and these functions as sort of ‘clock of life’ for each cell, counting down the limited replicative potential [23]. Telomere function is disturbed in both aging and cancer [24].

Telomeres are as such the protective repetitive nucleotide sequences located at the end of chromosomes. Since the DNA polymerase is incapable of fully replicating the termini of the DNA strands, each cell division results in a gradual shortening of the chromosome ends. Most somatic cells in eukaryotic organisms do not express telomerase, an enzyme that can elongate the telomeres and thus they will only be capable of a limited number of cell divisions before coding DNA gets compromised. Telomerase activity is high in both stem cells and cancer cells leading to the immortality of these cells. To avoid DNA instability in somatic cells, they will enter replicative senescence once telomeres shorten beyond a certain point and reach the Hayflick limit of cell division [23, 25]. The Hayflick limit (named after Leonard Hayflick), is based on the observation that human fetal cells in cell culture could only divide 40-60 times and then further cell division would stop. It has later been proven that the cause of this is the gradual shortening of the telomeres by each division until a critical limit where the cells cannot divide further without damaging coding DNA [25, 26]. The telomeres are also associated with protective proteins important in their regulation [27].

Telomere dynamics is an important factor in aging, age-related diseases and cancer. Telomere maintenance is determined by both inherited genetic factors and non-genetic factors such as stress, depression, smoking and exercise [27].

Inherited telomere syndromes where single gene inactivating mutations occur in telomere maintenance components are known. These mutations usually lead to short in vivo telomeres. Patients usually have phenotypes of accelerated aging such as diabetes, cardiovascular disease, hair graying and altered skin pigmentation in addition to loss of immune function and susceptibility to certain cancers [27, 28].

### Telomeres and aging

In the general population, short telomere length have been associated with many age-related diseases such as poor immune function [29], diabetes [30], cardiovascular disease [31, 32], osteoarthritis [33], atrial fibrillation [34] and Alzheimer’s disease [35]. Short telomere length is also associated with earlier death from age-related diseases in those over 60 [36], and overexpression of telomerase can inhibit aging but at the expense of increased tumorigenesis [37, 38]. Telomere length is also highly heritable and a study found that heritability counts for more than 50% of telomere length, and that sons have shorter telomeres than daughters at any given age. It showed that first-degree relatives of families with exceptional longevity and long telomeres had longer telomeres than second-degree relatives and also longer than their spouses [39].

### Telomeres and cancer

Telomerase upregulation in cancer cells is a pivotal event in cancer cells acquisition of a limitless replicative potential as one of the hallmarks of cancer. In 80-90% of fully malignant human tumors, the telomerase is upregulated compared with normal tissues [27]. This enzyme, human telomerase reverse transcriptase (hTERT) has the ability to build back on the cap ends that otherwise gets shortened by each cell division. By avoiding an ever-shorter cap end by hTERT, the cell avoids the Hayflick limit and does not enter apoptosis. Several studies and meta-analyses have examined the association of telomere length in blood leukocytes to cancer risk and mortality. Some studies showed that telomere attrition and short telomeres is a risk factor for cancer development [40-42]. The adjusted odds ratio (OR) for bladder cancer was 1.88 for individuals in the quartile with the shortest telomeres as compared with individuals in the quartile with the longest telomeres[40]. This also holds true for lung cancer (OR=2.39), smoking related cancers (OR=2.25), cancers of the digestive system (OR=1.69) and urogenital system cancers (OR=1.73). Incidence rates in a 10 year population follow up study was 5.1 per 1,000 in the longest telomere group, 14.2 in the middle group and 22.5 in the short telomere group [43]. Other studies show an association with cancer mortality [44-46]. Multivariable-adjusted hazard ratios of early death were 1.31 in individuals in the quartile and 1.43 in individuals in the decile with the shortest telomeres vs the longest [44], and in a large 7-year follow up study on 63,637 individuals the multivariable-adjusted hazard ratio of cancer mortality was 1.35 for individuals in the shortest vs the longest decile [46]. Thus, hTERT may act in some instances as a risk-factor for developing cancer, and in other setting as a driver for the aggressiveness of cancer. The association might be cancer-type specific and some cancers might be more dependent on telomerase than others. A meta-analyses showed stronger association between short telomeres and bladder, esophagus, gastric, head and neck, ovarian and renal cancers [47].

### Common role in aging and cancer

A recent study showed that age-related telomere attrition is faster in those who later developed cancer but that the attrition decelerates before cancer diagnoses resulting in longer telomeres 3-4 years prior to diagnoses [48]. This might represent telomere shortening being involved in early carcinogenesis followed by cancer hijacking and initiating methods of telomere elongation such as telomerase activation [48]. This might also affect blood leukocytes, which are major players in cancer development and progression. If this is validated in future studies, it may point to a potential as an early biomarker for cancer detection [48]. Therapeutic manipulation of hTERT may also have potential as a cancer-therapy. Further, how this can affect aging processes has been pursued also from a therapeutic perspective.

## 3. Epigenetic alterations

Epigenetics are changes in gene expression without altering the DNA sequence, such as DNA methylation, chromatin remodeling and non-coding DNA.

### DNA methylation

DNA methylation is a common epigenetic mechanism of gene regulation and promoter region hypermethylation is associated with gene silencing and hypomethylation is associated with increased gene transcription.

Methylation has been suggested as the predictor of human age in many genome wide methylation studies [49], but the relationship is complex. An increase in age is associated with increasing global hypomethylation, but many loci are also hypermethylated, such as many tumor suppressor genes [2, 50]. Cells from patients and mice with progeroid syndromes also demonstrate methylation patterns that mirror those found in normal aging [51]. A large meta-analysis of over 13,000 participants showed that DNA methylation-based biomarkers, also called "epigenetic age", predicts all cause mortality independent of chronological age, race and other risk factors. This was consistent over all the ethnic groups examined (Non-Hispanic whites, Hispanic and African American) [52]. "Epigenetic age" are robust estimators of chronological age described by Horvath [53] and Hannum [54] and is based on levels of methylation on a defined number of CpG dinucleotide markers [52].

Cancer cells are broadly characterized by hypermethylation and silencing of tumor suppressor genes including cell cycle regulators p21, p16^INK4a^, Rb, DNA repair genes exemplified by BRCA1 or receptors such as retinoic acid receptor (RARB) [55]. Cancer is also associated with hypomethylation and activation of proto-oncogenes and this seems to have a causative role in oncogenesis by causing chromosomal instability [56]. As an example the hypomethylation of specific long interspersed nuclear element-1 (LINE-1) is associated with activated proto-oncogenes MET, RAB3IP and CHRM3 in metastatic colorectal cancer [57], and hypomethylation and activation of the putative proto-oncogene ELMO3 is associated with lung cancer development and metastasis [58].

Aberrant methylation patterns are observed in almost all neoplasms and have potential as molecular markers in cancer prevention, prognosis and therapy [55]. Several natural compounds, such as resveratrol, green tea and curcumin, have shown potential in prevention and treatment of cancer through mechanisms of epigenetic alteration [59].

### Histone modification

Histones can be altered by covalent and reversible acetylations, methylations, sumolyation, ADP-ribosylation, ubiquitylation and phosphorylations [55]. This will alter the electric charge of amino residues on histones and make it bind the negatively charged DNA strands tighter or looser, resulting in actively transcribed loose euchromatin or the tighter silenced heterochromatin. These epigenetic changes also work in recruitment of the transcription regulation machinery [55]. The modifications can occur at many different spots on the histone tails and are controlled by numerous enzymes, including methyl transferases, demethylases, acetyltransferases, deacetylases. This regulation is very complex and involves many different players, but numerous studies link histone modification to both aging and cancer, and investigation is ongoing concerning drugs that can inhibit the enzymes involved. These drugs can potentially be used in both cancer therapy and to promote healthy aging. Analyses of large sets of sequenced DNA from tumors reveal that histone methyl transferases are frequently mutated in cancer, and represent 5% of driver genes identified in whole genome sequencing in breast cancer[60, 61]. This is also a potentially important target for drug innervation and many studies are ongoing [61].

Aging is also associated with specific complex alterations of histones and chromatin distribution. Histone-3 lysine-9 trimethylation (H3K9me3) is the hallmark of constitutive heterochromatin, and H3K9me2 is also heterochromatin associated. Aging is associated with a decrease or redistribution of the two with loss of repression over constitutive heterochromation loci and concomitant gain of facultative heterochromatin in other genomic regions [62]. H3Kme3 is associated with transcription start sites and is associated with active transcription, and elevated levels are associated with advanced age [62]. These are just a few examples of the novel and exciting field of histone modifications as related to aging. As we gain a better understanding of the field, drugs intervening with regulatory enzymes might be part of the drug arsenal of healthy aging in the future.

### Non-coding DNA

Non-coding DNA most importantly represented by microRNA also play an important role in gene regulation and emerging evidence suggest that they may regulate up to 80% of all expressed genes [63]. Evidence suggests that microRNA involvement in the regulation of many important pathways in both aging and cancer. For example, senescence, IGF-1 and mammalian target of rapamycin (mTOR) [49].

Aberrant expression of many different microRNA can contribute to both the development and prognosis of various cancer types [55]. As an example, many members of the let-7 family are down-regulated in breast cancer and this is associated with upregulation of estrogen receptors (ER). Experimental over-expression of let-7 in ER positive breast cancer tissue caused inhibition of cell proliferation and induction of apoptosis [55, 64].

The miR-17-92 cluster comprises 6 miRNAs which are implicated in both cancer and aging. The deletion of these is neonataly lethal in mice and they are regulators of development and aging. The expression is downregulated with age and overexpression is associated with life extension in mice [65]. The cluster is also protective against a variety of age related pathologies such as heart disease, neurodegenerative disease and osteoporosis [65]. A pleiotrophic role is suggested as it is involved in cells senescence and aging, but upregulation promotes oncogenic transformation and plasma levels are increased in a wide range of tumors suggesting a role as a potential alarm marker [65].

Antisense RNA can be used to interfere with miRNA and these have showed promise in oncological therapy of hepatocellular carcinoma, pancreatic cancer, glioblastoma and breast cancer in combination with radiochemotherapy [66].

CircularRNA (circRNA) are circular RNA sequences of approximately 100 nucleotides that have been known for a long time but little is known about their function. Emerging evidence shows that these RNA have important functions in a wide range of processes such as cell senescence, apoptosis, cell cycle regulation and aging among many others. They are also involved in cell signaling and can have a protective role in colorectal cancer [67] and expression is altered in many other malignancies [68].

## 4. *Loss of proteostasis*

Proteostasis is the homeostasis of our proteome; a healthy protein turnover. The proteostasis network consist of chaperone-mediated folding, proteasomal degradation and autophagy. Failure of proteostasis can lead to the accumulation and deposition of misfolded and proteo-toxic polypeptide aggregates compromising the vitality of the cell. This is the hallmark of many age-related diseases, especially neurodegenerative diseases such as Alzheimer’s or Parkinson’s disease, and also cataracts, the most common cause of blindness in the world which primarily affect the elderly [69, 70]. There is a progressive deterioration in proteostasis with age and almost all tissues of the aged organism will have a certain accumulation of protein aggregates and inclusions [71]. Proteostasis is also involved in cancer development and research into drugs targeting proteostasis is being investigated as potential new antineoplastic drugs and many have also entered the clinics.

### Molecular chaperones (heat shock proteins)

Chaperones and co-chaperones are evolutionary conserved small molecules that aid in the folding and refolding of polypeptides to functional proteins, or direct them to the degradation system if such folding cannot be accomplished. During evolution the chaperones also acquired extra chaperoning roles, such as immune system regulation, cell differentiation, gene expression, DNA replication, signal transduction, programmed cell death, cellular senescence and carcinogenesis [72]. The most important of these chaperones is the heat shock proteins (HSP) whose transcription is up-regulated during cellular stress.

Aging is associated with impaired induction of the chaperone system in response to stresses and as a result accumulation of misfolded proteins occur [73]. Overexpression of chaperones is associated with extended longevity in lab animals [2], so the induction of HSP could potentially halt aging by refolding the damaged accumulated proteins [74]. HSP levels are decreased with age in most tissues including neurons, and so is HSF (heat shock factor), the principal transcription factor for the heat shock proteins [74]. HSP are not only involved as a folding assistant, but can also guide proteins to the lysosomes in a process called chaperone mediated autophagy. This process is induced by starvation or oxidative stress [74]. This is believed to be one of the mechanisms by which starvation prolongs lifespan, by increasing protein turnover and causing damaged proteins to be degraded [75]. The dysregulation of the chaperone system with age is also proposed to cause dysregulation of the immune system as the two are tightly associated [76].

The chaperone system is also implicated in cancer development and progression, but instead of reduced activity such as in aging, over-activity seems to be the norm. The level of most heat shock proteins are significantly upregulated in most cancers tested, including colon, cervix and prostate [72]. Chaperones have been implicated in a range of human cancer types, being involved in various oncogenic processes such as cell proliferation, invasiveness, induction of neo-angiogenesis, the metastatic process, and in induction of immune tolerance [72]. This is achieved by mechanisms such as association with important signal transduction pathway members including HER2, ALK, EGFR and BRAF, the dysregulation of which is associated with several cancers [72, 77].

Chaperones can also bind and inactivate tumor suppressors (such as p53), stabilize VEGF and nitric oxide synthase involved in angiogenesis and can be secreted acting as signaling molecules [72]. Protection against tumor suppressive amyloidogenesis is also an important feature [78]. The over-expression of several HSPs is also associated with a poor prognosis in many cancers [72, 78].

HSPs can inhibit the induction of cellular senescence and apoptosis and, in effect, protect the cancer cells from chemotherapy [79]. As a result, their importance in cancer development and progression has led to a workload of research on chaperones as a target for cancer therapy.

Several inhibitors of HSP90 are currently in clinical trials and making their way into the clinics. These can be used in monotherapy or in combination with chemotherapy and have shown striking potential against subgroups of non-small cell lung cancer, and promising results in breast cancer, gastrointestinal stromal tumors (GISTs), melanoma and some hematological malignancies [80]. In preclinical trials the combination of a HSP90 inhibitor with radiotherapy shows great results in the usually radiochemotherapy resistant hepatocellular carcinoma[81]. HSP70 inhibitors have shown preclinical anti-cancer effects in multiple myeloma [82], and a HSP27 inhibitor has shown effect in castration resistant prostate cancer and is now undergoing phase 2 clinical trial [83]. Chaperone targeting drugs show promise as a new group within cancer therapy and a several avenues of research is currently ongoing.

**Figure 2.**
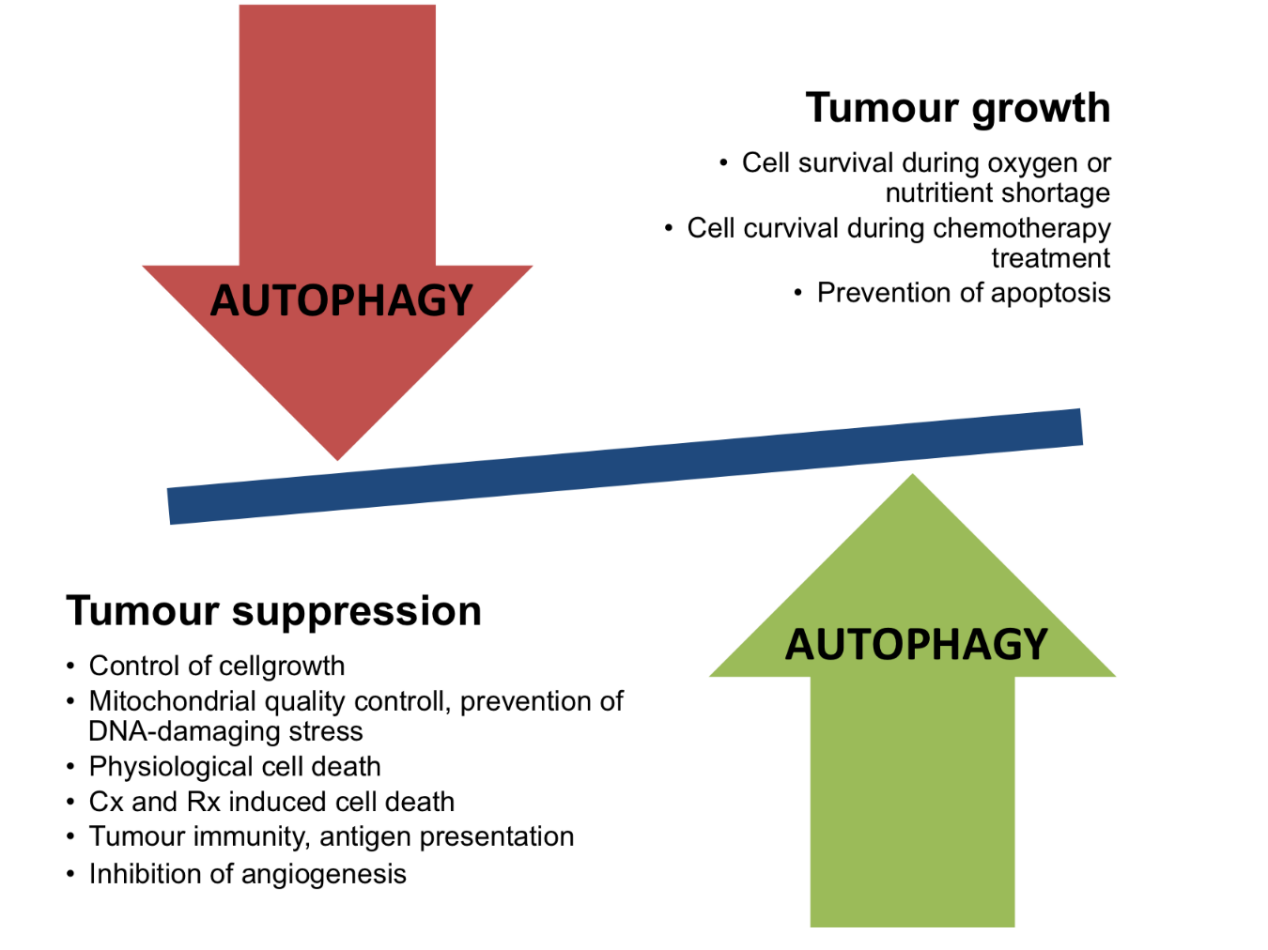
The dual role of autophagy in cancer Examples of mechanisms that are related to either tumor suppression or tumor growth where autophagy plays a role. Cx denotes chemotherapy, Rx denotes radioationtherapy.

### Proteasome

The ubiquitin-proteasome and autophagy-lysosome pathways are the main proteolytic systems and these are also essential for proteostasis. Proteasome activation delays aging in vivo and vitro in a variety of laboratory organisms [84]. Proteasome activity is also high in centenarians and has been postulated as one of the mechanisms for their healthy aging [85]. Compounds that increase proteasome activity such as dietary fatty acids, pollen, algae extract, spices and several synthetic compounds have resulted in prolonged and healthy living in laboratory animals and human cell lines [85].

Proteasome *inhibitors* have already made it to the clinics as treatment mainly for multiple myeloma and different lymphomas. Bortezomib (Velcade) was the first inhibitor approved in 2003, but several second generation inhibitors have emerged since Bortezomib has been a staple for multiple myeloma treatment at all stages of the disease and is commonly used in combination with strong glucocorticoids and chemotherapeutics [86]. Bortezomib is currently also used in mantle cell lymphoma and has shown good effects in many other lymphomas [86].

### Autophagy-lysosome

Autophagy is the lysosome-mediated way of degradation of misfolded proteins, damaged organelles and intracellular pathogens. Autophagy is the major intracellular degradation system by which cytoplasmic materials are delivered to and degraded in the lysosome. Although removal and degradation of cellular products is important, the sole purpose of autophagy is not the simple elimination of materials. Rather, autophagy also serves as a dynamic recycling system that produces new building blocks and energy for cellular renovation and homeostasis [87]. After autophagy, the resultant degradation products can be used for different purposes, such as new protein synthesis, energy production, and gluconeogenesis.

### Autophagy and aging - role of caloric restriction

Caloric restriction (usually by 20-40% of ad libitum intake) without malnutrition has long been one of the most established and potent ways of life extension and many of the mechanisms thought to mediate this effect converge on autophagy. Studies in mice have revealed that mice fed a 55-65% caloric restricted diet through their life exhibited a 35-65% greater mean and maximal lifespan than mice eating a non-purified-*ad libitum* diet [88, 89]. Prolonged caloric restriction has also been found to delay onset of age related diseases such as diabetes and cancer in calorie restricted rodents and non-human primates [88]. Caloric restriction in overweight humans has been shown to reduce cardiac risk factors, improve insulin sensitivity, enhance mitochondrial function and reduce oxidative damage do DNA [88]. It is still to early to conclude on a life extending effect in humans but evidence of the health benefits is emerging. Calorie restriction works by several mechanisms. It has antioxidative and anti-inflammatory actions through COX, NF-kB and MAPK pathway inhibition [90]. Calorie restriction also alters global DNA methylation and upregulates histone deacetylases among them the sirtuins, causing chromatin alterations [90]. The sirtuins also have effects through wide interaction with many transcription factors still not fully characterized [90]. Calorie restriction is also a potent way of inhibiting of cancer initiation and progression through the same pathways as above and also through a decrease in mTOR signaling, decreased growth factor signaling and decreased vascular perturbations [91]. Caloric restriction upregulates several signaling pathways that mediate increased lysosome-autophagy activity, including mTOR inhibition and sirtuin upregulation [92]. Autophagy is sufficient to extend lifespan in several laboratory animals including mice, and knockdown of several autophagy components abolishes this effect [93]. Autophagy gene polymorphism has been linked to age-related diseases such as osteoarthritis, senile osteoporosis and neurodegenerative disease [49]. Several compounds have been discovered to increase autophagy, extend lifespan and improve outcome in age related neurodegenerative diseases in model organisms. These include the mTOR inhibitor Rapamycin, the acetyltransferase inhibitor Spermidine and resveratrol, a compound isolated from the skin of red grapes and popularized as the central compound in the wine theory of the French paradox [49, 94].

### Autophagy and cancer - a dual role

Autophagy is upregulated and important for survival in most cancers, especially after treatment as most anticancer drugs will result in upregulation, giving rise to the theory that autophagy works as a way of chemo-resistance [95]. Autophagy works by degrading defective mitochondria and also through the degradation of fats and proteins providing the high energy requiring tumor cells with energy and glutamine [96]. Several lines of evidence suggest that autophagy has a dual role in carcinogenesis - on the one side through mechanisms that are promoting tumor growth, while on the other side inducing tumor suppression effects (Fig. 2). Knockout of autophagy in genetically engineered mouse models with NSCLC and pancreatic cancers leads to the transformation of malignant tumors to benign rare oncocytomas, tumors with accumulated defective mitochondria [96].

Since treatment with anticancer drugs upregulated autophagy many studies have been performed on cancer cell lines and experimental animals where chemotherapeutics has been given together with chloroquine or hydroxychloroquine, which are anti-malaria drugs used to inhibit autophagy. The combination shows increased cytotoxicity and apoptosis for the cancer cells in most cases [95]. Notably, these drugs might also be incorporated into the growing arsenal on antineoplastic drugs in the future.

## 5. Deregulated nutrient sensing and mitochondrial dysfunction

One of the earliest and most effective paths to extended longevity in a variety of laboratory animals including primates is calorie restriction, and nutrition and metabolism has long been connected to cancer as well. Common features of human aging includes increased visceral fat and decreased lean mass, insulin resistance, and increase in muscle ragged red fibers (accumulation of defective mitochondria within muscle cells) [49]. These features are related to diseases of old age, including cancer.

### Insulin like growth factor

Insulin like growth factor (IGF-1) is upregulated in response to growth hormone and signals through the same pathway as insulin. Together the signaling pathways are called IIS (insulin and IGF-1 signaling) pathway and is the most conserved aging controlling pathway in evolution and has many targets among them AKT, mTOR, RAS and FOXO [2, 97]. Genetic polymorphisms or mutations attenuating signaling at any level of this signaling pathway has been linked to increased longevity, and the pathway is linked to the beneficial effects of caloric restriction [98]. Insulin and IGF signaling has been shown to inhibit apoptosis and promote cell proliferation and division potentially extending the lifespan of genetically altered cells and has been linked to colorectal cancer, prostate cancer and breast cancer [97, 99]. Circulating levels of IGF1 and IGFBP3 has been linked to all cause mortality in men with advances prostate cancer and risk of developing prostate cancer [97]. Circulating levels of IGF1 and IGFBP3 have also been linked to all cause mortality and development of breast cancer in some reports, but studies are conflicting [97, 100]. High circulating IGF1 has also been linked to more locally advanced stages of colorectal cancer and less differentiated cases of colorectal cancer [97]. While an overall role in cancer appears present, the magnitude and specific contribution to cancer type sand stages remains uncertain.

### Diabetes

Diabetes mellitus type 2 is the number 3 risk factor for pancreatic cancer and is also linked to endometrial cancer [101, 102]. In a large meta analysis intentional weight loss with and without bariatric surgery was also linked to a significant reduction in cancer risk, but this was mostly attributed to a reduction in estrogen and pro-inflammatory cytokines, as the results for IGF were inconsistent [103]. Ongoing investigations look for the potential for IIS inhibiting drugs in cancer therapy with mixed results, as reviewed elsewhere [104].

### The mTOR and AMP Kinase (AMPK) pathways

Other important nutrient sensing pathway participants involved in both aging and cancer are mTOR and AMP Kinase (AMPK). The mTOR plays a key role in cellular metabolism, growth and proliferation [105]. Genetic down-regulation of the mTOR pathway extends longevity in a wide range of laboratory animals, and treatment with rapamycin in mice extends longevity and is considered the most robust chemical life extender in mammals [2]. Several mTOR inhibitors have entered the clinics primarily used as immunosuppressants and antineoplastic drugs. Up-regulated mTOR signaling is involved in the development, progression and metastasis of many different cancers including colorectal, prostate and breast cancer [105]. Temsirolimus, an mTOR inhibitor is currently used in treatment of advanced renal cell carcinoma and mantle cell lymphoma. Evrolimus is used in renal cell carcinoma, pancreatic neuroendocrine tumors, nonfunctional NET (neuroendocrine) tumors of gastrointestinal and lung origin [106], certain astrocytomas and hormone receptor positive advanced breast cancer. Studies investigating new indications are ongoing and second-generation mTOR inhibitors are underway. Metformin, a medicine commonly used in the treatment of diabetes mellitus type 2 activates AMP kinase, which causes inhibition of the mTOR pathway among other effects. Metformin inhibits proliferation of a wide range of cancer cell lines *in vitro* and significantly extends lifespan in various laboratory animals [2, 107, 108].

## 6. Cellular senescence

Cellular senescence is the process in which a cell irreversibly stops dividing and undergoes certain phenotypic alterations including chromatin changes and alterations in the secretome [49]. Cellular senescence can be triggered by various stress mechanisms such as telomere shortening, oncogene activation, DNA damage response (DDR) pathway and activation of p16^INK4a^. The critical executioners of cellular senescence seems to be Retinoblastoma 1 (Rb1) and p53, both of which are tumor suppressor proteins involved in carcinogenesis [109]. Data indicates that cells can memorize mitogenic stimuli and cell division history through progressive de-repression of CDKN2A and transcription of cell cycle inhibitors p16^INK4a^ and p19^ARF^, which ultimately leads to increased p53 and Rb1 causing cell senescence. This memory seems to involve epigenetic changes through Polycomb complexes and histone methylations [109].

Senescence works as an anticancer mechanism by inhibiting the proliferation of cells with telomere attrition and cells overexpressing oncogenes. This oncosuppressive effect is supported by the many senescent cells found in premalignant lesions [110]. This might be a protective effect early in life limiting the expansion of mutated and potentially harmful cell lines, but the accumulation of such senescent cells with age can be deleterious [111]. Senescence can also have tumor promoting effects [112], since senescent cells are also avid secretors of proinflammatorly cytokines and growth factors, known as the senescence associated secretory phenotype (SASP) and this secretion has been implicated in both cancer development and aging [49, 112]. Among these are proinflammatory factors TNF-α, IL-6, matrix metalloproteinases and monocyte chemoattractant protein-1, and growth factors such as FGF, EGF and VEGF [111, 113]. Inflammation is a well-known risk factor for many malignancies including tumorigenesis of the lung, initiated by pollution and smoke and perhaps involving miRNA regulation [114, 115]. Senescent cells are usually removed by immune surveillance and phagocytosis, but seem to gradually accumulate with age, most likely due to increased production as well as decreased elimination due to immunosenescence [49]. There is increasing evidence that senescence is involved in aging and as such aging might be the cost of cancer protection [112].

Both p16^INK4a^ and p53 are potent inducers of senescence and demonstrate antagonistic pleiotrophy in which protection against cancer leads to accelerated aging [112, 116]. p16^INK4a^ expression is markedly upregulated with age in humans and rodents and is a remarkably good biomarker of aging [109]. Mice lacking p16INK4a demonstrated higher regenerative capacity but at the expense of increased incidence of spontaneous and carcinogen induced cancers [112]. The INK4a/ARF locus was the genetic locus most strongly linked to age associated diseases in a genome wide association study [117], and conditional expression of p16^INK4a^ in mice lead to many of the physiological hallmarks of aging [118]. Removal of INK4a expressing senescent cells in mice delays onset and halts progression of aging and associated diseases [119].

## 7. Stem cells

Stem cells have an important role throughout our lives (Fig. 1), first during development and growth, and then in maintenance and repair during adult life. Stem cell depletion and dysfunction can give rise to many of the pathophysiological processes observed with aging, such as anemia and immunosenescence due to dysfunctional hematological stem cells [120], osteoporosis due to (loss of) mesenchymal stem cells [121] or sarcopenia due to (loss of) muscle stem cells [122]. Studies show that aging is accompanied with a diminished capacity to maintain homeostasis and to repair tissues after injury. When this ensues to the point that the tissues are no longer able to maintain homeostasis and adequate repair, physiologic decline and aging ensues [123].

Cancer is a disease associated with aging and the pathogenesis of cancer consists of multiple mutagenic events, and as such long lived stem cells seems to be the only cells capable of serving as such reservoirs. Stem cells represent the ideal cellular targets for the accumulation of precancerous lesions [123].

Cancer and aging might represent the two possible endpoints of a stem cells exposed to enough mutagenic hits (Fig. 1). Throughout life mutagenic hits will occur and if these cause genetic instability tumor suppressors such as p16^INK4a^ will cause cell cycle arrest, and apoptosis or senescence. If however the tumor suppressors fail, cancer may result [109]. In fact, hematopoetic stem cells of old mice undergo fewer divisions than those of young mice and this correlated with the accumulated DNA damage and expression of p16^INK4a^ [2, 124].

Stem cells also seems to mature and change quality as the organisms develop and hematological stems cells of young mice can give rice to different T and B cells subsets than old mice. The transcriptome is also fundamentally different between old and young cells. Lastly in young mice the lymphoid lineages predominate while this is skewed towards the myeloid lineage with age. This is also mirrored by the hematological malignancies in old and young [123].

### Conclusion

Aging is the inevitable path of life and cancer is usually thought of as an age-related disease, as cancer is increasingly common with advanced age and relatively rare in the young except leukemia. It is therefore not surprising that the two share many hallmarks. However, cancers cells and aged cells are also fundamentally opposite as cancer cells can be thought of as hyperactive cells with advantageous mutations, rapid cell division and increased energy consumption, while aged cells are hypoactive with accumulated disadvantageous mutations, cells division inability and a decreased ability for energy production and consumption. This is also reflected in the hallmarks of the two.

Aging and cancer share many hallmarks such as genomic instability, although cancer cells often benefit from mutations, other cells accumulate damaging mutations resulting in physiological decline and aging. Telomere attrition is also a common trait, but cancer cells circumvent cell cycle arrest by activating telomerase. Epigenetic changes are common in both and research and knowledge is accumulating at a rapid pace. There is a massive potential in inhibitors of enzymes responsible for the covalent modifications to DNA and histones, and also to antisense nucleic acid interfering with micro RNA.

Proteostasis is altered in both and protein aggregate accumulation and the toxic effects of it is the hallmark of many age-related diseases. Cancer avoids this by upregulating chaperone, proteasome and lysosome activity. Inhibitors of all pathways are already established in oncological treatment and new generations and new drugs are in continuous trials and development. Deregulated nutrient sensing is common to both and interference with the IIS pathway or mTOR pathway and AMP kinase pathway activation increase lifespan and halts cancer. Many mTOR pathway inhibitors have entered the clinics and metformin, a AMPK activator has potential both in cancer therapy and healthy aging.

Cell senescence is one of the hallmarks where cancer and aging are fundamentally different as accumulating DNA damage usually will cause a upregulation of cell cycle inhibitors leading to senescence or apoptosis while malignant cells avoid this by generating additional mutations such as deletion of tumor suppressors such as p16^INK4a^ or p53, an example of antagonistic pleiotrophy.

In summary, aging and cancer are interconnected in both time and mechanisms and many of the same strategies and drugs can be used to target both, while in other cases antagonistic pleiotrophy come in to effect and inhibition of one can be the activation of the other.
